# Fermented Ginger Extract in Natural Deep Eutectic Solvent Enhances Cytotoxicity by Inhibiting NF-κB Mediated CXC Chemokine Receptor 4 Expression in Oxaliplatin-Resistant Human Colorectal Cancer Cells

**DOI:** 10.3390/antiox11102057

**Published:** 2022-10-19

**Authors:** Ko-Chao Lee, Kuen-Lin Wu, Shun-Fu Chang, Hsin-I Chang, Cheng-Nan Chen, Yih-Yuan Chen

**Affiliations:** 1 Division of Colorectal Surgery, Department of Surgery, Kaohsiung Chang Gung Memorial Hospital, Kaohsiung 833, Taiwan; 2 Department of Medical Research and Development, Chang Gung Memorial Hospital Chiayi Branch, Chiayi 613, Taiwan; 3 Department of Biochemical Science and Technology, National Chiayi University, Chiayi 600, Taiwan

**Keywords:** colorectal cancer, oxaliplatin resistance, ginger extract, natural deep eutectic solvents, *Lactobacillus* fermentation

## Abstract

Ginger extracts have been shown to have health-promoting pharmacological activity and beneficial effects, including antioxidant and anticancer properties. The extraction of ginger by natural deep eutectic solvents (NaDES) has been shown to enhance bioactivity, but the cytotoxicity of NaDES extracts needs to be further determined. Signaling through the CXC chemokine receptor 4 (CXCR4) expressed on colorectal cancer (CRC) cells has a pivotal role in tumor cell chemosensitivity. Oxaliplatin is a third-generation platinum compound used as an effective chemotherapeutic drug for CRC treatment. However, whether ginger extract and oxaliplatin could induce a synergistic cytotoxic effect in oxaliplatin-resistant CRC cells through modulating CXCR4 expression is not known. In this study, oxaliplatin-resistant HCT-116 (HCT-116/R) cells were generated first. Ginger was extracted using the NaDES mixture betaine/lactate/water (1:2:2.5). *Lactobacillus reuteri* fermentation of NaDES-ginger extract increased the total polyphenol content (12.42 mg gallic acid/g in non-fermented NaDES-ginger extract and 23.66 mg gallic acid/g in fermented NaDES-ginger extract). It also increased the antioxidant activity by about 20–30% compared to non-fermented NaDES-ginger extract. In addition, it achieved low cytotoxicity to normal colonic mucosal cells and enhanced the anticancer effect on HCT-116/R cells. On the other hand, the inhibition of NF-κB activation by fermented NaDES-ginger extract significantly decreased the CXCR4 expression (*p* < 0.05) in HCT-116/R cells. The inactivation of NF-κB by pharmacological inhibitor pyrrolidine dithiocarbamate further enhanced the fermented NaDES-ginger extract-reduced CXCR4 expression levels (*p* < 0.05). Moreover, fermented NaDES-ginger extract could synergistically increase the cytotoxicity of oxaliplatin by inhibiting CXCR4 expression and inactivating NF-κB, resulting in HCT-116/R cell death. These findings demonstrate that fermented NaDES-ginger extract reduces the NF-kB-mediated activation of CXCR4 and enhances oxaliplatin-induced cytotoxicity in oxaliplatin-resistant CRC cells.

## 1. Introduction

Colorectal cancer (CRC) is the most commonly diagnosed gastrointestinal malignancy and the second leading cause of cancer-related mortality worldwide. Improvements in surgical techniques combined with effective chemotherapy strategies are currently the conventional therapy regimens for CRC patients [1]. Nevertheless, about 50% of CRC patients develop metastases and eventually have cancer recurrence [2]. Therefore, chemotherapy is still one of the choices for patients with CRC. Oxaliplatin is a platinum-based anticancer drug that induces apoptosis by disrupting DNA transcription and replication in tumor cells. However, the limitation to the effectiveness of CRC therapy lies in the development of intrinsic or acquired drug resistance to oxaliplatin therapy [3]. Oxaliplatin resistance in CRC is a major cause of treatment failure and results in poor prognosis in CRC patients; therefore, identification of the influencing signaling pathways and molecules associated with oxaliplatin resistance in cancer cells has an important role in the effective chemotherapy of CRC. It is necessary to investigate the therapy targets and develop strategies to enhance the chemosensitivity of oxaliplatin-resistant CRC cells.

Recently, a green solvent known as the natural deep eutectic solvents (NaDES) has been widely used to extract bioactive compounds from plant materials due to a wide range of advantages, including negligible volatility, non-flammability, biodegradability, sustainability, low toxicity, low cost, and easy production [4]. The strong hydrogen bonding between the extracted phytochemicals and the NaDES solutes increases the yield capacity and exhibits high solubility for different compounds, which is better than that of traditional organic solvents. *Zingiber officinale* Roscoe (commonly known as ginger) has special medicinal properties and has been shown to have beneficial pharmacological functions such as anti-bacterial, antioxidant, anti-inflammatory, anticancer, and antimetabolic syndrome activities [5]. The health benefits of ginger extract mainly come from its bioactive polyphenols, including gingerols and shogaols [6]. It has been reported that the antioxidant activity of ginger extracted by NaDES was higher than that extracted using organic solvent extract [7]. In addition, the NaDES mixture of betaine/lactate/water was found to be the effective solvent for ginger extraction, with increased total phenolic content and better antioxidant activity [8]. Nevertheless, the high viscosity of NaDES appears to increase the cytotoxicity of NaDES [9]. Organic acids have also been reported to contribute to the increased toxicity of NaDES mixtures [10].

The research on ginger extract in the chemoprevention of CRC has also received extensive attention. It has been shown that ginger extract could inhibit cancer cell proliferation and induce the apoptosis of multiple types of cancer in vitro [11]. However, there are no relevant published reports on the anticancer activity of NaDES processed ginger extract against chemotherapeutic drug-resistant CRC cells. C-X-C chemokine receptor type 4 (CXCR4) has been demonstrated to play a critical role in controlling the embryogenesis and normal cell functions [12]. Conversely, increased CXCR4 activities have been observed in many cancers, including CRC [12,13]. The overexpression of chemokine receptor CXCR4 in CRC cells has been shown to be associated with drug resistance and poor prognosis [13]. In oxaliplatin-resistant human CRC cells, the upregulation of CXCR4 has a critical role in the development of oxaliplatin-resistant characteristics [14]. The CXCR4-mediated signaling might have a specific role in regulating drug resistance mechanisms in CRC [15]. As a growing number of studies have demonstrated that the expression level of CXCR4 in CRC may influence the sensitivity of CRC to chemotherapeutic drugs such as oxaliplatin, the expression of CXCR4 in drug-resistant CRC cell lines for cancer chemotherapy has still been further elucidated. In here, we have evaluated the potential cytotoxicity of the fermented NaDES-ginger in the oxaliplatin-resistant CRC and examined the possible role of CXCR4 level and the underlying mechanism in this process.

## 2. Materials and Methods

### 2.1. Materials

The cell culture media, reagents (FBS and antibiotics), and all other cell culture materials were purchased from Gibco (Grand Island, NY, USA). The antibody against CXCR4 (PA5-19857) was purchased from Invitrogen (Carlsbad, CA, USA). The antibody against β-actin (#8457) and HRP–conjugate secondary antibody against rabbit IgG (#7074) was purchased from Cell Signaling Technology (Beverly, MA, USA). The ginger powder was obtained from Kemyth Biotech Co., Ltd., Taiwan. Pyrrolidine dithiocarbamate (PDTC), anhydrous betaine, D,L-lactic acid (80% aq. soln.), and all other chemicals of analytical grade were purchased from Sigma (St. Louis, MO, USA).

### 2.2. NaDES-Ultrasound-Assisted Extraction of Ginger Powder

NaDES were prepared from betaine and D, L-lactic acid, as previously described [8]. Briefly, anhydrous betaine was dissolved and mixed in D, L-lactic acid in a 1:2 molar ratio and placed in a round-bottom flask. The mixture was continuously stirred for 1 h at 60 °C until the NaDES formed a homogeneous liquid. Then, water (including water present in D- and L-lactic acid) was added to make the NaDES mixture into a betaine/lactate/water (1:2:2.5).

For NaDES-ultrasound extraction, ginger powder (10 g) was extracted with 150 mL of NaDES. The extraction conditions were set to 200 W at room temperature for 15 min. After the ultrasound extraction, the ginger extract was filtered with Whatman No. 1 filter paper, and the liquid was collected and then centrifuged at 5500× *g* rpm for 10 min at 4 °C. The supernatant of NaDES-ginger extract was subsequently filtered using a nylon membrane filter of 0.22 μm pore size and stored in the dark at 4 °C.

### 2.3. Fermentation of NaDES-Ginger Extract with L. reuteri

The *Lactobacillus* strain *L. reuteri* was tested and selected from the culture collection of probiotic strains at National Chiayi University, Taiwan. The NaDES-ginger extract was first adjusted to pH 6.0 by NaOH. The *L. reuteri* culture was prepared by culturing in MRS broth at 37 °C under anaerobic conditions. The inoculum concentration of *L. reuteri* was adjusted to 1 × 10^8^ CFU/mL, shaken at 100 rpm for 48 h, and fermented to the late stage of the stationary phase at 37 °C under the anaerobic condition. The solution of fermented NaDES-ginger extract was centrifuged for 20 min at 5000× *g* rpm, and the supernatants were collected. A stock solution of fermented NaDES-ginger extract (50 mg/mL) was then sterilized with a filter of 0.22 μm pore size, which was diluted with a serum-free cell culture medium before use. Since NaDES may not contain suitable carbon sources for *Lactobacillus* growth, we found that the fermentation of *L. reuteri* in NaDES without ginger extract did not increase the cell number of *L. reuteri*. In addition, the cytotoxicity effects of fermented NaDES, NaDES and culture medium only on HCT-116 cells were similar, and these control groups did not affect our experimental results. Therefore, the equivalent amount of fermented NaDES was taken as the control [16].

### 2.4. High-Performance Liquid Chromatography (HPLC) Analysis

The compounds of ginger extract were analyzed by HPLC (HITACHI, Primaide 1430 Diode Array Detector (DAD), Tokyo, Japan, Column: YMC-Triart C18, 250 mm 1 × 4.6 mm, 5 μM, Kyoto, Japan) consisting of a binary pump and an injector value with 20 μL loop. The temperature of the column was maintained at 40 °C. The mobile phase was water: methanol (20:80, *v*/*v*) for detecting 6-Shogaol (Figure S1). The flow rate was 1 mL/min for 20 min at a detection wavelength of 280 nm.

### 2.5. The DPPH and ABTS Antioxidant Assays

The percentage of the antioxidant activity of each sample was analyzed by 2,2-diphenyl-1-picrylhydrazyl (DPPH) and 2,2′-azino-bis (3-ethylbenzothiazoline-6-sulfonic acid) (ABTS) free radical assays. DPPH was dissolved in methanol for a DPPH reagent concentration of 80 μL/mL. Ginger extract samples in series of different concentrations were mixed with the DPPH reagent in methanol. After incubation, the absorbance was measured at 514 nm using the spectrophotometer; 100% methanol was used as a control. The scavenging activity percentage was measured using the formula: Inhibition (%) = [(A Control − A Sample)/A Control] × 100.

The ABTS radical was generated by 2 mM of ABTS solution and stored in the dark for 16 h. The samples were prepared by mixing 10 μL of each sample with 990 μL of ABTS; 100% methanol was used as blank. The ABTS solution (1 mL) was reacted with various concentrations of ginger extract, which was followed by measuring the absorbance at 734 nm using a spectrophotometer between 3 and 7 min. The following formula was used to assess the ABTS scavenging capacity: Inhibition (%) = [(A Control − A Sample)/A Control] × 100.

### 2.6. Total Phenolic Content (TPC) Analysis

TPC was determined using the Folin–Ciocalteu reagent [17]. Non-fermented and fermented NaDES-ginger extract (100 μL) were mixed with 500 μL Folin–Ciocalteu reagent and diluted with water (2.4 mL). Then, 2 mL of 7.5% sodium carbonate was added to the mixture and left for 1 h at room temperature. The absorbance was measured at 760 nm using a spectrophotometer. Gallic acid equivalents (GAE) were used to calculate the amount of TPC in milligrams per gram of the sample (mg GAE/g).

### 2.7. Cell Culture

NCM460, a cell line derived from normal colon mucosa, was obtained from INCELL Corporation (San Antonio, TX, USA) and incubated in M3F media supplemented with 10% FBS in a cell culture incubator. The CRC cell line HCT-116 was ordered from the cell bank of the Taiwan Food Industry Research and Development Institute (Hsinchu, Taiwan). HCT-116 cells were grown in DMEM supplemented with 10% FBS in a humidified 95% air and 5% CO_2_ condition and maintained at 37 °C. The oxaliplatin-resistant cell line HCT-116/R was established as previously described [14].

### 2.8. Cell Viability Assay

Cytotoxicity evaluation and cell growth inhibition were assessed with the MTT (3-(4,5-dimethylthiazol-2-yl)-2,5-diphenyltetrazolium bromide) assay, which is used to measure cellular metabolic activity as an indicator of cell viability and cytotoxicity. Normal NCM460 cells and CRC HCT-116/R cells with a density of 5 × 10^3^ cells per well were seeded in 96-well microplates. Different concentrations of ginger extract were added to 96-well plates after plating to evaluate cell viability. After treatment, the MTT solution (0.5 mg/mL) in serum-free medium was added to the wells and further incubated for 4 h at 37 °C. The purple formazan crystals were dissolved in DMSO, and the absorbance of the wells was measured immediately at 570 nm using a spectrophotometer [18].

### 2.9. Trypan Blue Staining Assay

Trypan blue solution is used to assess the proportion of cell death. After administration, cells were washed with PBS buffer followed by trypsin treatment. Cells were then harvested and centrifuged, and the cell pellet was resuspended in a complete medium. A 10 µL aliquot of the cell suspensions was mixed thoroughly with 0.4% trypan blue dye (90 μL), and the viable cells (unstained cells) and dead cells (stained cells) were counted using a hemocytometer. The proportion of dead cells was calculated as follows: trypan blue (+) cells ratio (%) = (stained cell number/total cell number) × 100.

### 2.10. Live/Dead Assay

A live/dead cell assay kit (L-3224 Invitrogen, Carlsbad, CA, USA) was used to analyze cell cytotoxicity after the treatment of HCT-116/R cells with fermented NaDES-ginger extract. Calcein AM is retained by viable cells and emits green fluorescence, whereas the ethidium homodimer generates red fluorescence in dead or damaged cells. Reactive dyes were prepared, and cells were stained according to the instructions in the kit and incubated at 37 °C for 30 min. Subsequently, the cells were observed by fluorescence microscopy.

### 2.11. Real-Time Quantitative Polymerase Chain Reaction (PCR)

The total RNA was isolated by the Trizol reagent. cDNA was synthesized from 1 μg of total RNA using a high-capacity reverse-transcription kit after the RNA purity and integrity were checked. A real-time PCR assay of the indicated genes was carried out using the SYBR Green Master Mix (Thermo, Waltham, MA, USA) in 96-well plates. The PCR primers were synthesized by PROtech Technology, Inc., Taipei, Taiwan. The following oligonucleotide primers of the indicated genes were used as previously described [19]: CXCR4 forward primer, 5′-ACTAC ACCGA GGAAA TGGGC T-3′, CXCR4 reverse primer, and 5′-CCCAC AATGC CAGTT AAGAA GA-3′; GAPDH forward primer, 5′-AGGTG AAGGT CGGAG TCAAC-3′; reverse primer, 5′-CCATG TAGTT GAGGT CAATG AAGG-3′). The GAPDH gene was employed as the internal control. All RT-PCR was determined in duplicate from three independent experiments. The relative expression of the target genes was calculated using the ΔΔCt method and normalized into GAPDH mRNA levels [18].

### 2.12. Western Blot Analysis

The cells were seeded overnight at a density of 1 × 10^6^ cells in 60 mm culture dishes before treatment. After administration, the cells were harvested, and the total protein was extracted with ice-cold lysis buffer (Thermo Scientific, Rockford, IL, USA). Protein concentration was quantified according to the Bradford protein assay kit (Bio-Rad, Hercules, CA, USA). Equal amounts of total protein lysates were loaded and separated by sodium dodecyl sulfate–polyacrylamide gel electrophoresis and blotted onto a nitrocellulose paper. The immunoreactive bands of designated primary antibodies were probed with secondary antibodies and detected with an enhanced chemiluminescence kit (The original, representative blots are shown in Figure S2).

### 2.13. siRNA Transfection

The CXCR4 siRNA (sc-35421) and non-targeting siRNA (sc-37007) were obtained from Santa Cruz Biotechnology (Santa Cruz, CA, USA). HCT-116 cells were incubated in a complete medium and transfected with 200 nM CXCR4 siRNA using the Lipofectamine RNAiMAX transfection reagent (Invitrogen, Carlsbad, CA, USA). CXCR4 siRNA transfection caused at least an 80% reduction in the respective mRNA and protein expression levels compared with the siRNA control vector (data not shown).

### 2.14. NF-κB p65 Transcription Factor Activity Assay

The nuclear extracts of the cells were isolated and collected by nuclear protein extract kits (Item No. 10009277, Cayman Chemical, Ann Arbor, MI, USA). The NF-κB p65 transcriptional activity by detecting specific nuclear DNA binding was determined with NF-κB (p65) Transcription Factor Assay kit (Item No. 10007889, Cayman Chemical, Ann Arbor, MI, USA). Equal amounts of nuclear proteins for each condition were analyzed according to the manufacturer’s protocol. Sample absorbances were measured at 450 nm by a microplate reader.

### 2.15. Statistical Analysis

Results are presented as mean ± standard error (S.E). The data were analyzed for significance by one-way analysis of variance (ANOVA) followed by Scheffe’s test for multiple comparisons. *p*-values less than 0.05 were considered significant.

## 3. Results

### 3.1. Fermentation Process to Changes of Chemical Compositions and Antioxidant Properties of Ginger Extract in NaDES

**Figure 1:**
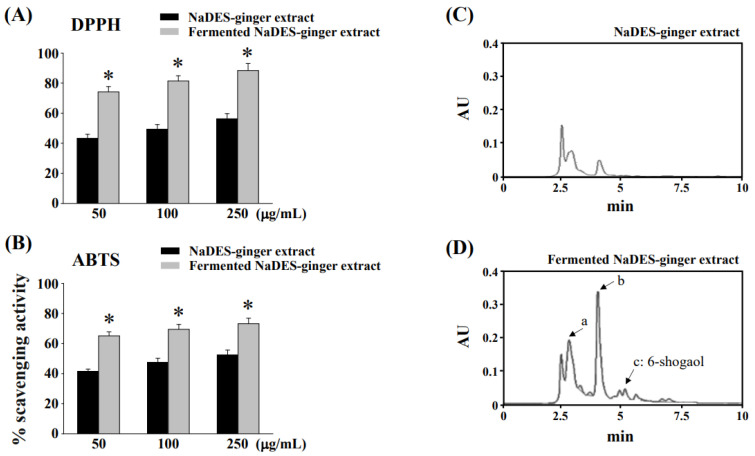
Changes in chemical compositions and antioxidant activity of the ginger extract in NaDES after fermentation. (**A**) DPPH radical scavenging activity and (**B**) ABTS radical scavenging activity of non-fermented NaDES-ginger extract and fermented NaDES-ginger extract. Bars represent the standard deviation from triplicate determination of each concentration. * *p* < 0.05 vs. non-fermented NaDES-ginger extract. HPLC profiles of (**C**) non-fermented NaDES-ginger extract and (**D**) fermented NaDES-ginger extract showing the main peaks increased (peaks a, b, and c) in fermented NaDES-ginger extract.

The antioxidant activity of NaDES-ginger extract before and after *L. reuteri* fermentation was determined using the DPPH and ABTS free radical scavenging assays. The DPPH and ABTS free radical scavenging activities of non-fermented and fermented NaDES-ginger extract with various concentrations were tested, as shown in Figure 1A,B, respectively. Our results showed that the fermented NaDES-ginger extract exhibited significantly higher DPPH and ABTS antioxidant activities than the non-fermented NaDES-ginger extract. In addition, as shown in the HPLC-based profile in Figure 1D, the compounds in the fermented NaDES-ginger extract were drastically changed compared to the non-fermented NaDES-ginger extract (Figure 1C).

The TPC in the ginger extract was reported to be related to its antioxidant capacity [20]. Therefore, we analyzed the effect of *L. reuteri* fermentation on TPC in non-fermented and fermented NaDES-ginger extract. The TPC of fermented NaDES-ginger extract (23.66 ± 0.34 mg of GAE/g) was found to be significantly increased (*p* < 0.05) compared to that of the non-fermented NaDES-ginger extract (12.42 ± 0.25 mg of GAE/g). Increased TPC in fermented NaDES-ginger extract may reflect improved antioxidant capacity, as shown in Figure 1A,B, and increased compound composition (peaks a, b, c), as shown in Figure 1D.

### 3.2. Cytotoxicity Evaluation of NaDES-Ginger Extract on Human Normal Colonic Mucosa Cell Line NCM460 after L. reuteri Fermentation

**Figure 2:**
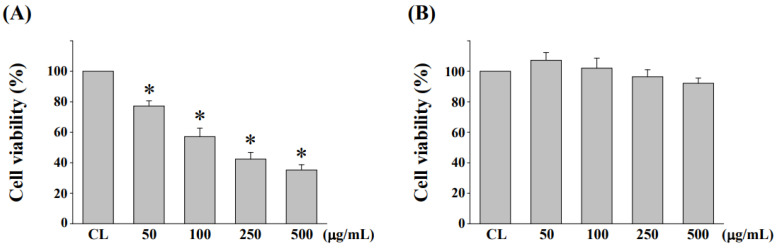
Evaluation of cytotoxicity of ginger extract in NaDES after fermentation. NCM460 cells were cultured in the presence of (**A**) non-fermented NaDES-ginger extract and (**B**) fermented NaDES-ginger extract for 24 h, and cell cytotoxicity was determined by MTT assay. Data represent the mean ± standard error of the mean from three independent experiments; * *p* < 0.05 vs. CL NCM460 cells.

The MTT assays were used to investigate the cytotoxicity effect on normal human mucosa cell line NCM460 of NaDES-ginger extract before and after *L. reuteri* fermentation. Treatments for the non-fermented NaDES-ginger extract at the concentration of 50 μg/mL or greater induced a significant decrease in cell viability (Figure 2A). After *Lactobacillus*-fermentation, the cytotoxicity of NaDES-ginger extract was completely changed, with no cytotoxicity up to 500 μg/mL (Figure 2B).

### 3.3. Cytotoxicity and CXC Chemokine Receptor 4 Expression of HCT-116 and Oxaliplatin-Resistant HCT116/R Cells

**Figure 3:**
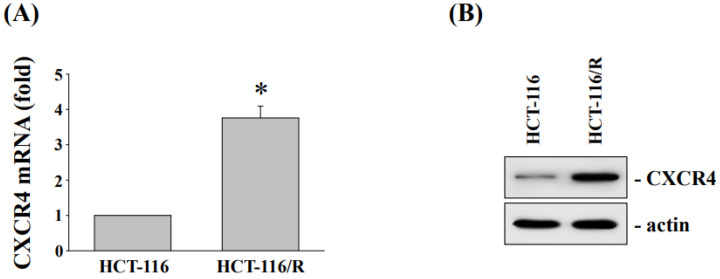
The CXCR4 expression of oxaliplatin-resistant CRC HCT-116/R cells and the parental cell line HCT-116. (**A**) Real-time polymerase chain reaction analysis of CXCR4 mRNA expression in HCT-116 and HCT-116/R cells. (**B**) Western blot analysis for the expression of CXCR4 in HCT-116 and HCT-116/R cells. * *p* < 0.01 vs. HCT-116/R cells.

To determine the mechanism underlying oxaliplatin resistance, oxaliplatin-resistant HCT-116 (HCT116/R) cells were created. Consistent with the previous research findings [14], the 50% inhibitory concentration values (IC50) of oxaliplatin in the HCT-116 and HCT-116/R cells were determined to be 4.7 μM and 24.5 μM, respectively. We also found that the expressions of CXCR4 mRNA (Figure 3A) and protein (Figure 3B) were significantly increased in HCT-116/R cells.

### 3.4. Fermented NaDES-Ginger Extract Decreased the Cell Viability of HCT-116/R Cells

**Figure 4:**
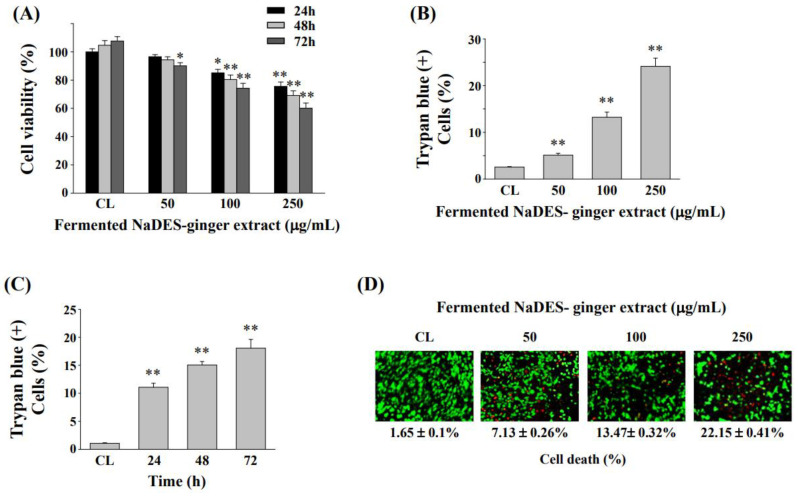
Cell survival of HCT-116/R cells treatment with fermented NaDES-ginger extract. (**A**) After starvation, HCT-116/R cells were treated with various concentrations of fermented NaDES-ginger extract (50–250 μg/mL) for 24, 48, and 72 h. Cell viability was determined by MTT assay. Cells were observed after the treatment with different concentrations of fermented NaDES-ginger extract for 24 h (**B**), and 100 μg/mL fermented NaDES-ginger extract for 24, 48, and 72 h (**C**). HCT-116/R cells were collected and stained with trypan blue dye, and the number of dead cells was manually counted. The percentage of trypan blue-positive cells represented the population of dead cells, and the mean ± standard error of the mean was from three independent experiments. * *p* < 0.05, ** *p* < 0.01 vs. control cells (cells treated with fermented NaDES without ginger extract). (**D**) HCT-116/R cells were treated with the indicated concentrations of fermented NaDES-ginger extract for 24 h. Cell death was determined by the live/dead cell viability assay.

Before *Lactobacillus* fermentation, the NaDES-ginger extract is significantly cytotoxic to normal human mucosa cells (Figure 2A). In order to avoid cytotoxicity caused by NaDES-ginger extract, we only used fermented NaDES-ginger extract to analyze the anticancer effect on HCT-116/R cells. After incubating HCT-116/R cells with vehicle (fermented NaDES) or different concentrations of fermented NaDES-ginger extract for 24–72 h, the cell viability was determined by MTT assay and expressed as a percentage relative to the control group, which was set as 100%. As shown in Figure 4A, the fermented NaDES-ginger extract decreased the HCT-116/R cell viability in both time- and dose-dependent manner.

The Trypan blue staining assay also showed that fermented NaDES-ginger extracts enhanced the HCT-116/R cell death in a dose-dependent manner (Figure 4B). In addition, fermented NaDES-ginger extracts at 100 μg/mL were observed to increase HCT-116/R cell death in a time-dependent manner (Figure 4C). The live/dead cell viability assay further confirmed the dose-dependent effect on the induction of cell death in the HCT-116/R cells by fermented NaDES-ginger extract treatment (Figure 4D).

### 3.5. Downregulation of CXCR4 mRNA and Protein Levels in HCT-116/R Cells after Treatment by Fermented NaDES-Ginger Extract

**Figure 5:**
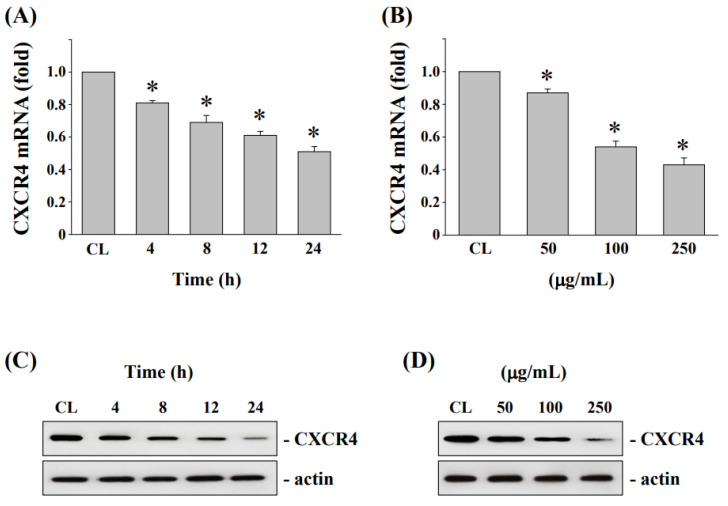
Fermented NaDES-ginger extract decreased the CXCR4 expression in a dose and time-dependent manner. (**A**) HCT-116/R cells were cultured in a complete medium and then exposed to fermented NaDES-ginger extract (100 μg/mL) for 4, 8, 12, or 24 h. (**B**) Various concentrations of fermented NaDES-ginger extract (50–250 μg/mL) were added to cells for 24 h in the complete medium. The total RNA was isolated and examined by real-time polymerase chain reaction for CXCR4 mRNA expression. Data represent the mean ± standard error of the mean from three independent experiments. * *p* < 0.05 vs. control cells. (**C**,**D**) After treatment as above, the cell extracts were examined by Western blot for determination of CXCR4 protein levels. Results in (**C**,**D**) are representative of three independent experiments with similar results.

To determine whether CXCR4 expressions are associated with the anticancer effect by fermented NaDES-ginger extract administration, HCT-116/R cells were treated with the fermented NaDES-ginger extract (100 μg/mL) for 4–24 h or at various concentrations (50, 100, 250 μg/mL) for 24 h. The CXCR4 mRNA and protein expression levels in HCT-116/R cells were evaluated by real-time PCR and Western blot analysis, respectively. The results show that the decreased CXCR4 mRNA (Figure 5A) and protein (Figure 5C) expressions were time-dependent in HCT-116/R cells by the fermented NaDES-ginger extract treatments. In addition, the decreased expression of CXCR4 mRNA and protein in HCT-116/R cells after treatment with 100 μg/mL fermented NaDES-ginger extract treatment was dose-dependent (Figure 5B,D).

### 3.6. Down-Regulation of CXCR4 Expression Enhanced Cell Cytotoxicity and Cell Death in HCT-116/R Cells

**Figure 6:**
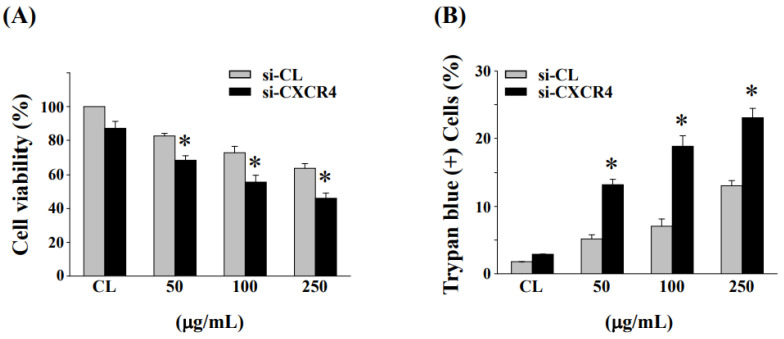
Downregulation of CXCR4 expression enhanced the fermented NaDES-ginger extract-induced cytotoxicity and cell death. (**A**) HCT-116/R cells were transfected with siRNA against CXCR4 (si-CXCR4) or scrambled (si-CL) in the complete medium for 24 h prior to the treatment with fermented NaDES-ginger extract (50–250 μg/mL). Cytotoxicity was determined by (**A**) MTT assay and (**B**) trypan blue exclusion assay. The results (mean ± standard error of the mean) were from three independent experiments. * *p* < 0.05 vs. si-CL-transfected cells.

Next, we examined the effect of CXCR4 gene knockdown by specific CXCR4 siRNA on fermented NaDES-ginger extract-induced cell cytotoxicity and cell death in HCT-116/R cells. The inhibition of CXCR4 expression by si-CXCR4 exhibited increased cytotoxicity to fermented NaDES-ginger extract treatment as compared to control siRNA transfected cells (Figure 6A). Moreover, the downregulation of CXCR4 expression could enhance the fermented NaDES-ginger extract-induced death of HCT-116/R cells (Figure 6B).

### 3.7. Inhibition of NF-κB Activation Enhances the Reduction in CXCR4 Expression in HCT-116/R Cells

**Figure 7:**
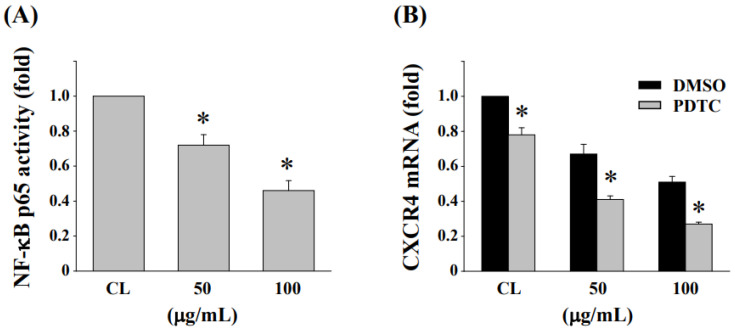
Fermented NaDES-ginger extract decreased CXCR4 expression via NF-κB inactivation in HCT-116/R cells. (**A**) HCT-116/R cells were cultured in a complete medium, and then, 50 or 100 μg/mL of fermented NaDES-ginger extract were added to the cells and incubated for 24 h. The NF-κB p65 activity was analyzed by enzyme-linked immunosorbent assay. The results (mean ± standard error of the mean) were from three independent experiments. * *p* < 0.05 vs. control cells. (**B**) NF-κB inhibitor pyrrolidine dithiocarbamate (PDTC; 10 μM) was added to HCT-116/R cells for 1 h before fermented NaDES-ginger extract treatment for a further 24 h. The results (mean ± standard error of the mean) were from four independent experiments. * *p* < 0.05 vs. cells treated with DMSO.

HCT-116/R cells were treated with fermented NaDES-ginger extract (50 and 100 μg/mL) for 24 h, and then, the activation of NF-κB was analyzed by NF-κB transcription factor activity assays. Cells treated with fermented NaDES-ginger extract resulted in a significant decrease in NF-κB p65 activity compared to the untreated control (Figure 7A). To determine whether CRCR4 expression in HCT-116/R cells is regulated by NF-κB, cells were pretreated with DMSO or PDTC, a specific inhibitor for NF-κB, for 1 h and then treated with fermented NaDES-ginger extract (50 and 100 μg/mL) for 24 h. Pretreating HCT-116/R cells with NF-κB inhibitor enhanced the expression of fermented NaDES-ginger extract-reduced CXCR4 mRNA as compared with DMSO-pretreated cells (Figure 7B).

### 3.8. Fermented NaDES-Ginger Extract and Oxaliplatin Exert a Synergistic Cytotoxic Effect on HCT-116/R Cells

**Figure 8:**
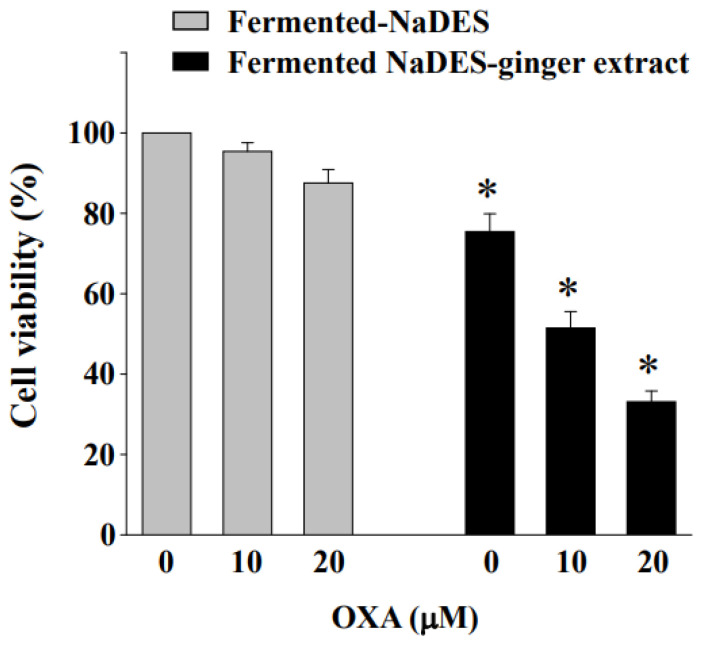
Fermented NaDES-ginger extract enhanced the reduction in cell viability in oxaliplatin-exposed HCT-116/R cells. HCT-116/R cells were cultured in a complete medium and then treated with fermented NaDES-ginger extract (100 μg/mL) and oxaliplatin (10 and 20 μM) for 24 h. After treatment, cell cytotoxicity was determined by MTT assay. The results (mean ± standard error of the mean) were from three independent experiments. * *p* < 0.05 vs. control cells (cells treated with fermented NaDES without ginger extract.

We sought to determine whether the fermented NaDES-ginger extract could enhance the cytotoxic effect of oxaliplatin by inhibiting the expression of CXCR4 in HCT-116/R cells. The MTT assays were used to examine the synergistic effect of combined treatments on cell viability. According to Figure 8, the combined treatment for 24 h with fermented NaDES-ginger extract (100 μg/mL) and oxaliplatin (10 and 20 μM) led to an increased reduction in cell viability in HCT-116/R cells compared to the control cells (co-treated with NaDES and oxaliplatin).

## 4. Discussion

CRC is one of the most commonly diagnosed malignancies and causes of cancer deaths around the world. The development of resistance to chemotherapeutic drugs during cancer treatment limits the effectiveness of cancer therapy; thus, it is crucial to develop potential strategies to overcome drug-resistant CRC cells [21]. The aims of this study were to explore the roles of CXCR4 in oxaliplatin-resistant CRC cells and the mechanism underlying the regulation of CXCR4 expression. We also investigated the anticancer activity of the newly designed fermented NaDES-ginger extract and further elucidated its role in the treatment effectiveness of the oxaliplatin-resistant CRC cell line. Fermented NaDES-ginger extract was then combined with the chemotherapeutic drug oxaliplatin to analyze the efficacy of the drug combination. We created oxaliplatin-resistant cells in CRC cell line HCT-116 (HCT-116/R cells) and found a higher level of CXCR4 expression in HCT-116/R cells than in the parental cells. We also found that NF-κB signaling is involved in the CXCR4 expression in HCT-116/R cells. The fermented NaDES-ginger extract exerted an effective anticancer activity against HCT-116/R cells through the downregulation of NF-κB activation and CXCR4 expression. Compared to using a chemotherapeutic drug alone, the combination with oxaliplatin showed enhanced anticancer activity on drug-resistant CRC cells.

Due to the insufficiency of traditional organic solvents and water, the development of green solvents is a priority for the application in plant extraction techniques [22]. NaDES is a new class of green chemical solvent consisting of at least one hydrogen bond acceptor and one hydrogen bond donor with a wide range of applications in organic synthesis, electrochemistry, plant extraction media, biosynthesis, and separation processes, enzymatic reactions or other biomedical applications [23]. However, the use of NaDES in medicinal plant extraction must be controlled and studied. A previous study showed that NaDES, comprised of betaine and glycerol, improved the extraction efficiency of chlorogenic acid from green coffee beans. However, the oral administration of 3 mL NaDES-green coffee bean extract by gavaged for 14 days resulted in the death of two out of six exposed rats, while no deaths occurred in the control group (six rats gavaged with 3 mL of water). In addition, weight loss, hepatomegaly, and elevated plasma oxidative stress levels were also observed [24]. Natural plant materials contain a variety of biologically active compounds or antioxidants, which have anti-aging, anticancer, anti-inflammatory, and other biological activities. However, it is unstable and sometimes irritating after extraction, implying toxicity to the human body [25]. Therefore, reducing toxicity or converting these phytochemicals into stable derivatives to increase their effectiveness requires further study. Furthermore, it has been reported that the transformation of phytochemicals by probiotics can lead to various consequences, such as increased bioavailability, improved pharmacological effects, and reduced toxicity [26]. Based on a previous study [8], we directly selected betaine/lactate/water as the NaDES solvent for ginger extraction in this study. The cytotoxic effect of different concentrations of NaDES-ginger extract before and after *L. reuteri* fermentation was determined on a normal human mucosa cell line NCM460 cells. We herein showed a dramatic change of functional properties of NaDES-ginger extract from the compositional as well as the antioxidant aspect. In addition, after *Lactobacillus* fermentation, the cytotoxicity of NaDES-ginger extract was completely decreased. Therefore, careful selection of ingredients and how they are used is necessary when using NaDES as a solvent for plant extraction.

The bioconversion of bioactive compounds by *Lactobacillus* fermentation was recently demonstrated to exhibit enhanced anticancer and antioxidant activities [16,27]. Strains of *L. rhamnosus* have been used earlier for the biotransformation of various glycosylated phytochemicals [28,29]. The use of *Lactobacillus* fermentation can also raise the rate of bioconversion to bioactive metabolites and give rise to the release of aglycones [30]. Various phenolic compounds in plants can be converted into aglycone forms, which have higher antioxidant and anticancer activities [31,32]. The phenolic compounds are major components of ginger extracts, which can inhibit carcinogenesis and mutagenesis through antioxidant activity [33]. Studies have also found that the TPC of many plant extracts can be significantly increased after Lactobacillus fermentation, which is accompanied by an increase in antioxidant capacity [34,35]. The results of the present study also found that the NaDES-ginger extract significantly increased TPC and antioxidant capacity after *L. reuteri* fermentation.

Ginger extract has been shown to modulate a variety of signaling molecules or up- or downregulate gene expression in cancer cells, including transcription factors, inflammatory mediators, protein kinases, drug resistance proteins, and chemokine receptors [11]. A previous study also reported that ginger extract could enhance the anticancer effect of the chemotherapeutic drug 5-fluorouracil in addition to inhibiting the carcinogenesis of CRC cells [36]. In this study, we conducted the fermentations using our *L. reuteri* strain. This fermentation product showed significantly increased anticancer activity in oxaliplatin-resistant CRC cells. Our results showed that inhibiting CXCR4 expression by a fermented NaDES-ginger extract enhanced cytotoxicity in oxaliplatin-resistant HCT-116/R cells. Liu et al. reported that elevated NF-κB activity can lead to increased chemoresistance in cancer cells and also found a correlation between NF-κB activation and drug resistance-induced tumor progression [37]. In neuroblastoma, the expression status of NF-κB-p65 was positively correlated with the expression level of CXCR4 [38]. Furthermore, NF-κB could activate CXCR4 expression through binding to the CXCR4 promoter, subsequently leading to increased migration and metastasis of cancer cells [39]. The evaluation of the fermented NaDES-ginger extract and the possible mechanism—by which its combination with oxaliplatin promotes the death of drug-resistant CRC cells—demonstrates the importance of the biotransformation of ginger extract for its anticancer effects through fermentation. Our study revealed that increased CXCR4 expression in oxaliplatin-resistant CRC cells was mediated via NF-κB activation. Although the correlation between CXCR4 expression and CRC resistance was not precisely quantified, the results shown in Figure 3 confirmed that oxaliplatin-resistant CRC cells have a significantly increased expression of CXCR4. In addition, the stimulation of fermented NaDES-ginger extract on HCT-116/R cells dramatically decreased the expression of CXCR4 (Figure 5), while the siRNA-mediated inhibition of CXCR4 gene expression would further enhance the lethal effect of fermented NaDES-ginger extract on CRC cells (Figure 6). Our results suggested that differences in the degree of CXCR4 expression could influence the susceptibility of drug-resistant CRC cells to chemotherapeutic agents and hence the eventual virulence effects. Thus, CXCR4 and NF-κB signaling may be effective targets for drug delivery and the development of anticancer therapies for drug-resistant CRC cells.

## 5. Conclusions

Our study demonstrates that fermented NaDES-ginger extract augments the cytotoxic and therapeutic effects of oxaliplatin in oxaliplatin-resistant CRC cells through the suppression of NF-κB and CXCR4. The results of this study suggest that the pharmacological effects of chemosensitivity in drug-resistant CRC cells may be enhanced by inhibiting CXCR4 expression. Further research is needed to examine the function of possible metabolites in ginger extracts after fermentation. In addition, the role of fermented NaDES-ginger extracts and other chemotherapeutic drugs and their combination under chemoresistant conditions in vivo also requires further exploration. Nonetheless, the combination of a fermented NaDES-ginger extract with oxaliplatin appears to offer a therapeutic strategy for treating chemoresistance in CRC.

## Data Availability

Data are contained within the article and Supplementary Material.

## Supplementary Materials

The following supporting information can be downloaded at: https://www.mdpi.com/article/10.3390/antiox11102057/s1, Figure S1: The HPLC profile for 6-shogaol standard; Figure S2: Original, representative blots corresponding to figures in this study.

## References

[1] Malki A., ElRuz R.A., Gupta I., Allouch A., Vranic S., Al Moustafa A.E. (2020). Molecular Mechanisms of Colon Cancer Progression and Metastasis: Recent Insights and Advancements. Int. J. Mol. Sci..

[2] Filip S., Vymetalkova V., Petera J., Vodickova L., Kubecek O., John S., Cecka F., Krupova M., Manethova M., Cervena K. (2020). Distant Metastasis in Colorectal Cancer Patients-Do We Have New Predicting Clinicopathological and Molecular Biomarkers? A Comprehensive Review. Int. J. Mol. Sci..

[3] Zhou J., Kang Y., Chen L., Wang H., Liu J., Zeng S., Yu L. (2020). The Drug-Resistance Mechanisms of Five Platinum-Based Antitumor Agents. Front. Pharmacol..

[4] Ling J.K.U., Hadinoto K. (2022). Deep Eutectic Solvent as Green Solvent in Extraction of Biological Macromolecules: A Review. Int. J. Mol. Sci..

[5] Mao Q.Q., Xu X.Y., Cao S.Y., Gan R.Y., Corke H., Beta T., Li H.B. (2019). Bioactive Compounds and Bioactivities of Ginger (Zingiber officinale Roscoe). Foods.

[6] Arcusa R., Villaño D., Marhuenda J., Cano M., Cerdà B., Zafrilla P. (2022). Potential Role of Ginger (Zingiber officinale Roscoe) in the Prevention of Neurodegenerative Diseases. Front. Nutr..

[7] Hsieh Y.H., Li Y., Pan Z., Chen Z., Lu J., Yuan J., Zhu Z., Zhang J. (2020). Ultrasonication-assisted synthesis of alcohol-based deep eutectic solvents for extraction of active compounds from ginger. Ultrason. Sonochem..

[8] Tzani A., Kalafateli S., Tatsis G., Bairaktari M., Kostopoulou I., Pontillo A.R.N., Detsi A. (2021). Natural Deep Eutectic Solvents (NaDESs) as Alternative Green Extraction Media for Ginger (Zingiber officinale Roscoe). Sustain. Chem..

[9] Hayyan M., Mbous Y.P., Looi C.Y., Wong W.F., Hayyan A., Salleh Z., Mohd-Ali O. (2016). Natural deep eutectic solvents: Cytotoxic profile. Springerplus.

[10] Lomba L., Ribate M.P., Sangüesa E., Concha J., Garralaga M.P., Errazquin D., García C.B., Giner B. (2021). Deep Eutectic Solvents: Are They Safe?. Appl. Sci..

[11] Lechner J.F., Stoner G.D. (2019). Gingers and Their Purified Components as Cancer Chemopreventative Agents. Molecules.

[12] Kawaguchi N., Zhang T.T., Nakanishi T. (2019). Involvement of CXCR4 in Normal and Abnormal Development. Cells.

[13] Nengroo M.A., Maheshwari S., Singh A., Verma A., Arya R.K., Chaturvedi P., Saini K.K., Singh A.K., Sinha A., Meena S. (2021). CXCR4 intracellular protein promotes drug resistance and tumorigenic potential by inversely regulating the expression of Death Receptor 5. Cell Death Dis..

[14] Huang W.S., Hsieh M.C., Huang C.Y., Kuo Y.H., Tung S.Y., Shen C.H., Hsieh Y.Y., Teng C.C., Lee K.F., Chen T.C. (2016). The Association of CXC Receptor 4 Mediated Signaling Pathway with Oxaliplatin-Resistant Human Colorectal Cancer Cells. PLoS ONE.

[15] Goïta A.A., Guenot D. (2022). Colorectal Cancer: The Contribution of CXCL12 and Its Receptors CXCR4 and CXCR7. Cancers.

[16] Lee K.C., Wu K.L., Yen C.K., Chang S.F., Chen C.N., Lu Y.C. (2022). Inhibition of NLRP3 by Fermented Quercetin Decreases Resistin-Induced Chemoresistance to 5-Fluorouracil in Human Colorectal Cancer Cells. Pharmaceuticals.

[17] Singleton V.L., Orthofer R., Lamuela-Raventos R.M. (1999). Analysis of total phenols and other oxidation substrates and antioxidants by means of Folin-Ciocalteau reagent. Method. Enzymol..

[18] Lee K.C., Wu K.L., Yen C.K., Chen C.N., Chang S.F., Huang W.S. (2021). 6-Shogaol Antagonizes the Adipocyte-Conditioned Medium-Initiated 5-Fluorouracil Resistance in Human Colorectal Cancer Cells through Controlling the SREBP-1 Level. Life.

[19] Huang W.S., Chen C.N., Sze C.I., Teng C.C. (2013). Visfatin induces stromal cell-derived factor-1 expression by β1 integrin signaling in colorectal cancer cells. J. Cell. Physiol..

[20] Ali A.M.A., El-Nour M.E.M., Yagi S.M. (2018). Total phenolic and flavonoid contents and antioxidant activity of ginger (Zingiber officinale Rosc.) rhizome, callus and callus treated with some elicitors. J. Genet. Eng. Biotechnol..

[21] Van der Jeught K., Xu H.C., Li Y.J., Lu X.B., Ji G. (2018). Drug resistance and new therapies in colorectal cancer. World J. Gastroenterol..

[22] Hikmawanti N.P.E., Ramadon D., Jantan I., Mun’im A. (2021). Natural Deep Eutectic Solvents (NADES): Phytochemical Extraction Performance Enhancer for Pharmaceutical and Nutraceutical Product Development. Plants.

[23] Liu Y., Friesen J.B., McAlpine J.B., Lankin D.C., Chen S.N., Pauli G.F. (2018). Natural Deep Eutectic Solvents: Properties, Applications, and Perspectives. J. Nat. Prod..

[24] Benlebna M., Ruesgas-Ramón M., Bonafos B., Fouret G., Casas F., Coudray C., Durand E., Cruz Figueroa-Espinoza M., Feillet-Coudray C. (2018). Toxicity of Natural Deep Eutectic Solvent Betaine:Glycerol in Rats. J. Agric. Food Chem..

[25] Klaschka U. (2015). Naturally toxic: Natural substances used in personal care products. Environ. Sci. Eur..

[26] Son C.G., Lee S.K., Choi I.K., Jang E.S., Bang K.J. (2020). Herbal Transformation by Fermentation. J. Acupunct. Meridian Stud..

[27] Kuo H.C., Kwong H.K., Chen H.Y., Hsu H.Y., Yu S.H., Hsieh C.W., Lin H.W., Chu Y.L., Cheng K.C. (2021). Enhanced antioxidant activity of Chenopodium formosanum Koidz. by lactic acid bacteria: Optimization of fermentation conditions. PLoS ONE.

[28] Kwon J.E., Lee J.W., Park Y., Sohn E.H., Choung E.S., Jang S.A., Kim I., Lee D.E., Koo H.J., Bak J.P. (2018). Biotransformation of Pueraria lobata Extract with Lactobacillus rhamnosus vitaP1 Enhances Anti-Melanogenic Activity. J. Microbiol. Biotechnol..

[29] Hati S., Vij S., Singh B.P., Mandal S. (2015). β-Glucosidase activity and bioconversion of isoflavones during fermentation of soymilk. J. Sci. Food Agric..

[30] Zhao Y.S., Eweys A.S., Zhang J.Y., Zhu Y., Bai J., Darwesh O.M., Zhang H.B., Xiao X. (2021). Fermentation Affects the Antioxidant Activity of Plant-Based Food Material through the Release and Production of Bioactive Components. Antioxidants.

[31] Leonard W., Zhang P., Ying D., Adhikari B., Fang Z. (2021). Fermentation transforms the phenolic profiles and bioactivities of plant-based foods. Biotechnol. Adv..

[32] Ryu J.Y., Kang H.R., Cho S.K. (2019). Changes Over the Fermentation Period in Phenolic Compounds and Antioxidant and Anticancer Activities of Blueberries Fermented by Lactobacillus plantarum. J. Food Sci..

[33] de Lima R.M.T., Dos Reis A.C., de Menezes A.P.M., Santos J.V.O., Filho J.W.G.O., Ferreira J.R.O., de Alencar M.V.O.B., da Mata A.M.O.F., Khan I.N., Islam A. (2018). Protective and therapeutic potential of ginger (Zingiber officinale) extract and [6]-gingerol in cancer: A comprehensive review. Phytother. Res..

[34] Aprile A., Negro C., Sabella E., Luvisi A., Nicolì F., Nutricati E., Vergine M., Miceli A., Blando F., De Bellis L. (2019). Antioxidant Activity and Anthocyanin Contents in Olives (cv Cellina di Nardò) during Ripening and after Fermentation. Antioxidants.

[35] Alharbi H.F., Algonaiman R., Barakat H. (2022). Ameliorative and Antioxidative Potential of Lactobacillus plantarum-Fermented Oat (*Avena sativa*) and Fermented Oat Supplemented with Sidr Honey against Streptozotocin-Induced Type 2 Diabetes in Rats. Antioxidants.

[36] Prasad S., Tyagi A.K. (2015). Ginger and its constituents: Role in prevention and treatment of gastrointestinal cancer. Gastroenterol. Res. Pract..

[37] Liu T., Wei R., Zhang Y., Chen W., Liu H. (2019). Association between NF-κB expression and drug resistance of liver cancer. Oncol. Lett..

[38] Zhi Y., Lu H., Duan Y., Sun W., Guan G., Dong Q., Yang C. (2015). Involvement of the nuclear factor-κB signaling pathway in the regulation of CXC chemokine receptor-4 expression in neuroblastoma cells induced by tumor necrosis factor-α. Int. J. Mol. Med..

[39] Singh A., Srivastava N., Yadav A., Ateeq B. (2020). Targeting AGTR1/NF-κB/CXCR4 axis by miR-155 attenuates oncogenesis in glioblastoma. Neoplasia.

