# Supplementary material for: Fermented Ginger Extract in Natural Deep Eutectic Solvent Enhances Cytotoxicity by Inhibiting NF-κB Mediated CXC Chemokine Receptor 4 Expression in Oxaliplatin-Resistant Human Colorectal Cancer Cells
Source: Antioxidants (Basel). 2022 Oct 19;11(10):2057. doi: 10.3390/antiox11102057 (PMC9598626; doi:10.3390/antiox11102057)

Figure S1. The HPLC profile for 6-shogaol standard.

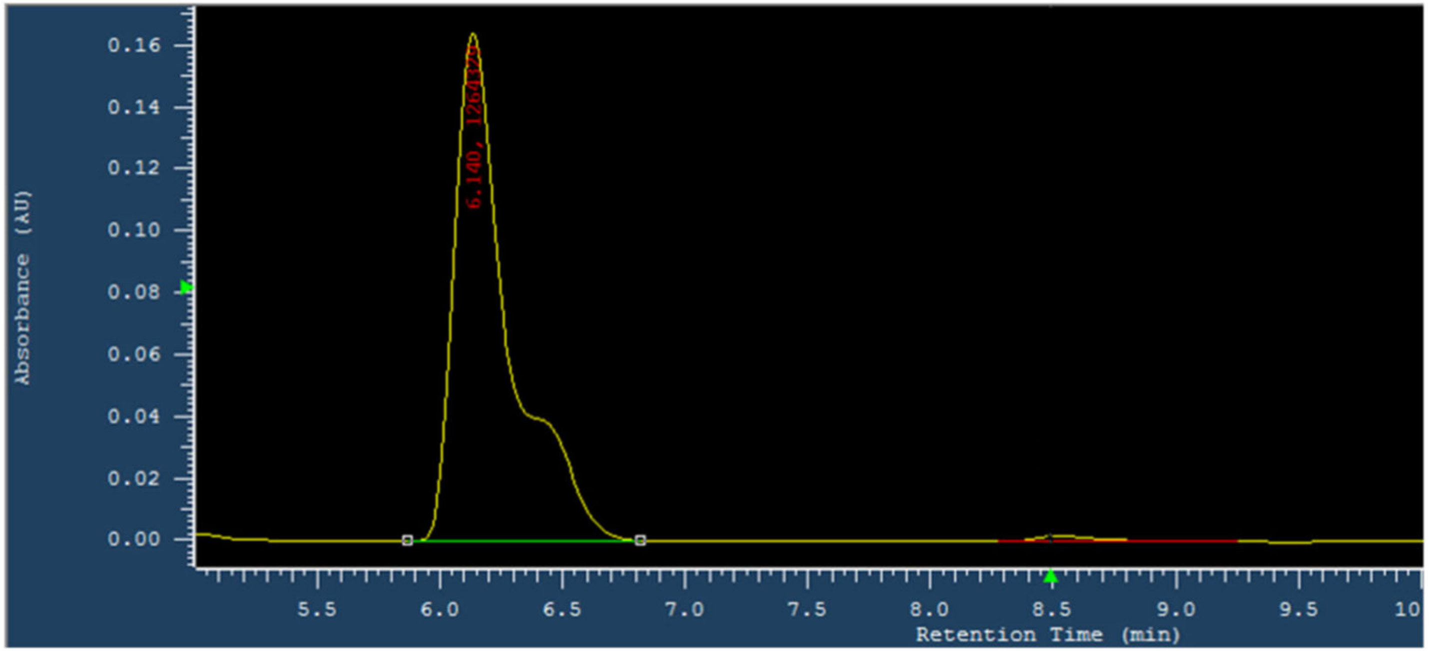

Figure S2. Original, representative blots corresponding to Figures in this study.

Figure 2C

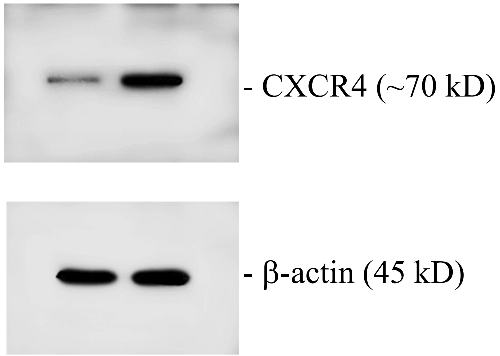

Figure 4C

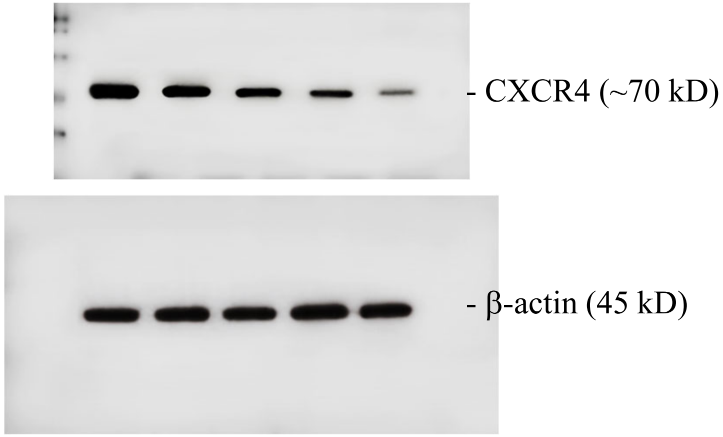

Figure 4D

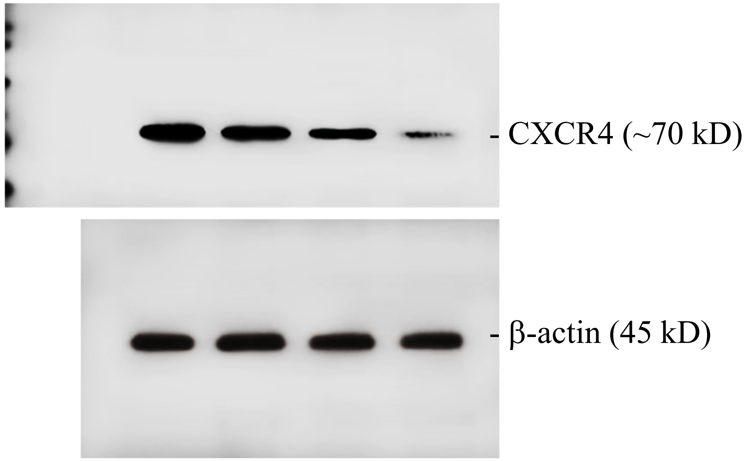

Supplement: Supplementary file 1 [file antioxidants-11-02057-s001.zip › antioxidants-1898320-supplementary.pdf]
